# Empagliflozin Contributes to Polyuria via Regulation of Sodium Transporters and Water Channels in Diabetic Rat Kidneys

**DOI:** 10.3389/fphys.2019.00271

**Published:** 2019-03-19

**Authors:** Sungjin Chung, Soojeong Kim, Mina Son, Minyoung Kim, Eun Sil Koh, Seok Joon Shin, Seung-Hyun Ko, Ho-Shik Kim

**Affiliations:** ^1^Division of Nephrology, Department of Internal Medicine, College of Medicine, The Catholic University of Korea, Seoul, South Korea; ^2^Department of Biochemistry, College of Medicine, The Catholic University of Korea, Seoul, South Korea; ^3^Division of Endocrinology, Department of Internal Medicine, College of Medicine, The Catholic University of Korea, Seoul, South Korea

**Keywords:** sodium-glucose cotransporter-2, empagliflozin, diuresis, sodium transport, water channel

## Abstract

Besides lowering glucose, empagliflozin, a selective sodium-glucose cotransporter-2 (SGLT2) inhibitor, have been known to provide cardiovascular and renal protection due to effects on diuresis and natriuresis. However, the natriuretic effect of SGLT2 inhibitors has been reported to be transient, and long-term data related to diuretic change are sparse. This study was performed to assess the renal effects of a 12-week treatment with empagliflozin (3 mg/kg) in diabetic OLETF rats by comparing it with other antihyperglycemic agents including lixisenatide (10 μg/kg), a glucagon-like peptide receptor-1 agonist, and voglibose (0.6 mg/kg), an α-glucosidase inhibitor. At 12 weeks of treatment, empagliflozin-treated diabetic rats produced still high urine volume and glycosuria, and showed significantly higher electrolyte-free water clearance than lixisenatide or voglibose-treated diabetic rats without significant change of serum sodium level and fractional excretion of sodium. In empagliflozin-treated rats, renal expression of Na^+^-Cl^-^ cotransporter was unaltered, and expressions of Na^+^/H^+^ exchanger isoform 3, Na^+^-K^+^-2Cl^-^ cotransporter, and epithelial Na^+^ channel were decreased compared with control diabetic rats. Empagliflozin increased an expression of aquaporin (AQP)7 but did not affect AQP3 and AQP1 protein expressions in diabetic kidneys. Despite the increased expression in vasopressin V2 receptor, protein and mRNA levels of AQP2 in empagliflozin-treated diabetic kidneys were significantly decreased compared to control diabetic kidneys. In addition, empagliflozin resulted in the increased phosphorylation of AQP2 at S261 through the increased cyclin-dependent kinases 1 and 5 and protein phosphatase 2B. These results suggest that empagliflozin may contribute in part to polyuria via its regulation of sodium channels and AQP2 in diabetic kidneys.

## Introduction

A recent trial with empagliflozin, a selective sodium-glucose cotransporter type 2 (SGLT2) inhibitor, has shown the significant improvement in the primary major adverse cardiovascular event outcome, driven mainly by significant reductions in cardiovascular death and hospitalization for heart failure (Zinman et al., 2015; Heerspink et al., 2016; van Bommel et al., 2017). In addition, empapliflozin-treated patients had a significant reduction in new-onset or worsening of nephropathy (Wanner et al., 2016). Considering that the small reduction in hemoglobin A1c level in the trial are unlikely to explain the rapid onset and effect size (van Bommel et al., 2017), hemodynamic effects such as a reduction in effective circulating volume load are a more likely mechanism of benefit observed with empagliflozin (Perkins et al., 2016). Accordingly, one of the most possible mechanisms for the positive cardiovascular and renal outcomes with empagliflozin would relate to effects on osmotic diuresis and natriuresis (Perkins et al., 2016; Rajasekeran et al., 2016; Reed, 2016).

Surprisingly, clinical and experimental data relating to diuresis or natriuresis after use of empagliflozin are scarce. Empagliflozin treatment over 28 days in patients with type 2 diabetes did not result in clinically relevant and significant changes in urine volume although there was a trend of increase in 24-h urine volume after even day 1 of administration (Heise et al., 2013). An 8-week clinical trial in type 1diabetics showed that subjects with normal filtration of kidney did not have the significant increased 24-h urine volume but there was a significant rise in 24-h urine volume in individuals with renal hyperfiltration (Cherney et al., 2014). Natriuresis is dose-dependently induced by another SGLT2 inhibitor, dapagliflozin, during the first 24 h in healthy volunteers, but returns to baseline after 13 days of intervention (Komoroski et al., 2009). A previous study also showed that urinary Na excretion tended to increase on day 1 after administration of canagliflozin but returned to baseline from day 2 to 5 (Tanaka et al., 2017). Experimental data using streptozotocin-induced diabetic rats showed that acute blockade with dapagliflozin increased Na and chloride excretion three-fold, whereas chronic blockade, defined by administrating dapagliflozin twice daily, did not significantly affect Na or chloride excretion (Thomson et al., 2012). The dapagliflozin treatment during 7 and 14 days showed the significantly decreased urine volume and the increased urine osmolality, but there was no significant change in the expression of aquaporin (AQP)2 and Na^+^-K^+^-2Cl^-^ cotransporter (NKCC2) proteins in the diabetic rat kidneys between dapagliflozin-treated and untreated diabetic rats (Chen et al., 2016). Thus, it is not clear how more prolonged use of a SGLT2 inhibitor would affect natriuresis and diuresis.

This study was designed to investigate and compare the effects of 12-week treatment with empagliflozin, lixisenatide, a glucagon-like peptide receptor (GLP)-1 agonist, and voglibose, an α-glucosidase inhibitor on renal tubular function, related Na transporters and water channels in diabetic rats.

## Materials and Methods

### Animal Experiments

Male 6-week-old Otsuka Long-Evans Tokushima Fatty (OLETF) and Long-Evans Tokushima Otsuka (LET) rats (Central Lab. Animal Inc., Seoul, Republic of Korea) were housed and cared individually for in an animal facility under controlled conditions (12/12 h light/dark cycle), and were given free access to chow and water during the whole study period. Body weight was measured every week. To assess the effect of empagliflozin on Na and water transport in renal tubules, we compared empagliflozin (3 mg/kg/day, given by dissolving in drinking water; Boehringer Ingelheim Pharma GmbH & Co KG, Germany) with two other antidiabetic agents: lixisenatide (10 μg/kg/day, intraperitoneally; Sanofi-Aventis Co., LTD, France), a GLP-1 agonist, and voglibose (0.6 mg/kg/day, given by dissolving in drinking water; Sigma-Aldrich, St. Louis, MO, United States), an α-glucosidase inhibitor. Each agent or vehicle was administered from 14 weeks of age to one of the following five groups (*n* = 8, each): non-diabetic LETO control group (LETO), diabetic OLETF control group (OLETF_C), empagliflozin-treated diabetic OLETF group (OLETF_E), lixisenatide-treated diabetic OLETF group (OLETF_L), and voglibose-treated diabetic OLETF group (OLETF_V). At the end of the 12-week experimental period, a 24-h urine sample, blood, and kidneys were obtained. The quickly removed kidneys were stored in 10% buffered formalin or frozen in liquid nitrogen and kept at -70°C for further analyses. The experiments were approved by the Institutional Animal Care and Use Committee of The Catholic University of Korea Seoul St. Mary’s Hospital.

### Biochemical Measurements

Fasting blood glucose level was measured weekly with an Accu-Chek meter (Roche Diabetes Care, Inc., Indianapolis, IN, United States). For 24-h urine collection, animals were housed individually in metabolic cages (Tecniplast S.p.A., Castronno, Italy). Measurement of blood and urine levels of glucose, creatinine, calcium (Ca), phosphate (P), Na, potassium (K), and osmolality was performed using enzymatic colorimetric methods (Modular DPP system, Roche, Hamburg, Germany). As prescribed previously (Hong et al., 2014), creatinine clearance (CCr) was calculated by a standard formula: CCr = urine creatinine (mg/dL) × urine volume (mL/24 h)/serum creatinine (mg/dL) × 1440 (min/24 h). Free water clearance (FWC) and electrolyte-free water clearance (EFWC) were also calculated as previously described (Veeraveedu et al., 2008):

$$FWC=\text{urine volume}(mL/24\;h)-[\text{urine osmolality}(\text{mOsmol/}H_{2}Okg)/\text{serum osmolality}\left(\text{mOsmol/}H_{2}Okg\right)]\times \text{urine volume}\left(mL/24\;h\right)$$

EFWC =urine volume(mL/24 h)−[urine Na(mmol/L)+urine K(mmol/L)]×urine volume(mL/24 h)/serum Na(mmol/L)

The factional excretion (Fe) of Na, K, Ca, and P was calculated using the following formula (Musso et al., 2012):

Feα=[urine  α(mmol/L)×serum creatinine(mg/dL)/serum α(mmol/L)×urine creatinine(mg/dL)]×100(α:Na, K, Ca, orP)

Transtubular K gradient (TTKG) was calculated as follows (Chung et al., 2010):

$$TTKG=[\text{urine K}\left(mmol/L\right)\times \text{serum osmolality}\left(\text{mOsmol}/H_{2}Okg\right)]/\left[\text{serum K}\left(\text{mmol/L}\right)\times \text{urine osmolality}\left(\text{mOsmol/}H_{2}Okg\right)\right].$$

### Western Blot Analysis

Proteins for western blot analysis were extracted from the kidney tissues using a PRO-PREP Protein Extraction Kit (iNtRON Biotechnology, Seongnam, Republic of Korea) according to the manufacturer’s instructions. Protein concentration was measured by Bradford assay (Bio-Rad Laboratories, Inc., Hercules, CA, United States). Equal protein samples were loaded on sodium dodecyl sulfate-polyacrylamide gel electrophoresis gels and transferred to nitrocellulose (NC) membranes. Membranes were incubated with the following primary antibodies: AQP1 (Alomone Labs, Jerusalem, Israel), AQP2 (Alomone Labs), p261-AQP2 (Novus Biologicals, Littleton, CO, United States), AQP3 (Alomone Labs), AQP7 (Alomone Labs), cyclin-dependent kinase 1 (cdk1; Santa Cruz Biotechnology, Inc., Santa Cruz, CA, United States), cdk5 (Santa Cruz Biotechnology, Inc.), Na+/H+ exchanger isoform 3 (NHE3; StressMarq Biosciences Inc., Victoria, BC, Canada), extracellular signal–regulated kinase (ERK; Cell Signaling Technology, Danvers, MA, United States), p-Erk (Cell Signaling Technology), *p*-glycogen synthase kinase 3α (pGSK3α; Santa Cruz Biotechnology, Inc.), Na+-Cl- cotransporter (NCC; StressMarq Biosciences), NKCC2 (StressMarq Biosciences), phosphatase 1β (PP1β; Santa Cruz Biotechnology, Inc.), PP2B (Santa Cruz Biotechnology, Inc.), vasopressin V2 receptor (V2R; Alomone Labs), α-epithelial Na+ channel (ENaC; StressMarq Biosciences); γ-ENaC (StressMarq Biosciences), p38 mitogen-activated protein kinase (p38; Cell Signaling Technology), p-p38 (Cell Signaling Technology) and β-actin (Sigma-Aldrich, St. Louis, MO, United States). After washing, the membranes were incubated with horseradish peroxidase-linked IgG. The protein bands were detected by enhanced chemiluminescence and imaged using an Image Quant LAS 4000 (GE Healthcare, Piscataway, NJ, United States). Densities of protein bands were determined by Quantity One 1-D analysis software (Bio-Rad Laboratories, Inc.) with normalization to those of the respective β-actin bands in the same samples.

### Quantitative Real-Time Polymerase Chain Reaction

Total RNA of the kidney tissues was extracted with TRIzol Reagent (Thermo Fisher Scientific, Waltham, MA, United States) according to the manufacturer’s instructions. For quantitative real-time polymerase chain reaction (qRT-PCR), SYBR Premix (Takara Bio Inc., Shiga, Japan) was used in an ABI PRISM 7900HT Sequence Detection System (Applied Biosystems, Foster City, CA, United States). Primer sequences for each gene were as follows: AQP1-sense (CTG CTG GCC ATT GAC TAC ACT G), anti-sense (GGT TTG AGA AGT TGC GGG TGA G); AQP2-sense (CAT GTC TCC TTC CTT CGA GCT G), anti-sense (CCC CAC GGA TTT CTA CTG GAG T); AQP3-sense (AGC AGA TCT GAG TGG GCA GT), anti-sense (CTT GGG CTT AAG AGG GGA AC); NKCC2-sense (CGG GTC GTC TAG ATC CAA AA), anti-sense (ATG GAC TTG GAA ACG ACT GG); and V2R-sense (GCT CTT CAT CTT TGC TCA GCG T), anti-sense (TCC AGG TGA CAT AGG CAC GAA). Each sample was run in triplicate. The mRNA level of each gene was normalized by the mRNA level of glyceraldehyde-3-phosphate dehydrogenase in the same tissue, and its relative changes among samples were calculated by the ΔΔCt method (Schmittgen and Livak, 2008) with the normalized mRNA level of each gene in non-diabetic LETO control rats set to one fold.

### Immunohistochemistry and Immunofluorescence

The 4-μm-thick sections of kidneys were deparaffinized, hydrated and incubated with 3% H_2_O_2_ in methanol followed by blocking with 10% normal goat serum in PBS. The sections were incubated with anti-AQP1 (Alomone Labs), anti-AQP2 (Alomone Labs), anti-AQP3 (Alomone Labs), anti-NCC (StressMarq Biosciences), anti-NKCC2 (StressMarq Biosciences), anti-NHE3 (StressMarq Biosciences) or anti-SGLT2 (Abcam, Cambridge, United Kingdom) antibodies overnight at 4°C. Antibodies were then localized through incubation with a peroxidase-conjugated horse anti-rabbit IgG for 1 h at room temperature and DAB substrate solution using the Vector Immpress kit (Vector Laboratories, Inc., Burlingame, CA, United States). The sections were dehydrated in ethanol, cleared in xylene and mounted without counterstaining. The slides were examined in a blinded-manner using Olympus BX-50 light microscopy (Olympus, Tokyo, Japan). For quantification of the proportional area of staining, more than 20 images per each slide were captured and analyzed to determine positive area using ImageJ software 1.49 (National Institutes of Health, Bethesda, MD, United States).

For immunofluorescence, the deparaffinized kidney sections were incubated with primary antibody against AQP2 (Alomone Labs), followed by nuclear staining with 4′,6-diamidino-2-phenylindole. After washing with PBS, the sections were incubated with Alexa 488-conjugated anti-rabbit IgG. The slides were mounted in Vectashield (Vector Laboratories) and sealed with a coverslip. The immunofluorescence signal was visualized and photographed with LSM 700 laser scanning microscope (Carl Zeiss MicroImaging GmbH, Jena, Germany).

### Statistical Analysis

Statistical analyses were performed using SPSS version 19.0 (IBM SPSS, Armonk, NY, United States). All values are expressed as mean ± standard error of the mean. Continuous variables between groups were compared by a one-way analysis of variance, followed by a Bonferroni *post hoc* test. A *p*-value < 0.05 represented statistically significance.

## Results

### Effects of Empagliflozin, Lixisenatide, and Voglibose on Renal Biochemical and Functional Parameters

Table 1 presents body weight and blood and urine biochemical data. At the end of treatment, the body weight of diabetic OLETF rats treated with antidiabetic agents tended to be lower than that of untreated OLETF rats, but which was not significant. Treatment with empagliflozin, lixisenatide, or voglibose in OLETF rats significantly decreased blood glucose, and no differences were observed in blood sugar levels among the diabetic OLETF rats treated with antidiabetic agents. In empagliflozin-treated OLETF rats, urinary glucose excretion was significantly higher than those of LETO or voglibose-treated OLETF rats.

**Table 1:** Body weight, blood, and urine measurements.

	LETO (*n* = 8)	OLETF_C (*n* = 8)	OLETF_E (*n* = 8)	OLETF_L (*n* = 8)	OLETF_V (*n* = 8)
Body weight (g)	560.1 ± 9.4	559.9 ± 16.9	523.2 ± 19.9	508.3 ± 26.3	534.8 ± 49.4
**Blood measurements**	
Glucose (mg/dL)	359.4 ± 22.8	731.1 ± 27.1^A^	541.5 ± 31.5^B^	586.1 ± 24.2^C^	521.8 ± 73.3^D^
Osmolality (mOsmol/kg H_2_O)	307.6 ± 1.4	329.9 ± 3.2^E^	321.0 ± 3.0^F^	320.9 ± 1.4^G^	319.8 ± 3.6^H^
Na (mmol/L)	137.3 ± 0.7	133.3 ± 0.9^I^	136.8 ± 0.9	138.8 ± 0.6	138.8 ± 1.1
K (mmol/L)	5.6 ± 0.3	5.7 ± 0.3	5.9 ± 0.2	5.0 ± 0.1	5.2 ± 0.4
Ca (mg/dL)	10.0 ± 0.0	10.2 ± 0.2	10.4 ± 0.1	10.3 ± 0.1	10.6 ± 0.2^J^
P (mg/dL)	5.5 ± 0.3	6.5 ± 1.5	5.1 ± 0.5	5.2 ± 0.4	5.4 ± 0.6
Creatinine (mg/dL)	0.42 ± 0.02	0.38 ± 0.05	0.33 ± 0.02	0.33 ± 0.01	0.39 ± 0.06
**Urine measurements**	
Glucose excretion (g/day/100 g body weight)	0.01 ± 0.0	13.25 ± 1.07^K^	10.3 ± 2.1^L^	7.5 ± 1.9^M^	3.0 ± 1.1
Na excretion (mmol/day/100 g body weight)	0.14 ± 0.02	0.32 ± 0.02^N^	0.26 ± 0.09	0.24 ± 0.04	0.16 ± 0.02^O^
K excretion (mmol/day/100 g body weight)	0.28 ± 0.04	0.59 ± 0.04^P^	0.59 ± 0.09^Q^	0.40 ± 0.05	0.31 ± 0.18
Ca excretion (mg/day/100 g body weight)	1.6 ± 0.2	5.3 ± 0.5	5.0 ± 0.6	5.2 ± 1.3	21.8 ± 4.6^R^
P excretion (mg/day/100 g body weight)	12.1 ± 2.5	27.3 ± 3.6	39.0 ± 5.8^S^	20.5 ± 2.9	19.8 ± 7.6
Creatinine excretion (mg/day/100 g body weight)	0.28 ± 0.01	0.20 ± 0.01^T^	0.22 ± 0.02	0.25 ± 0.01	0.19 ± 0.02^U^


*^*A*^*P* < 0.001 vs. LETO; ^*B*^*P* = 0.045 vs. LETO and *P* = 0.041 vs. OLETF_C; ^*C*^*P* < 0.001 vs. LETO and *P* = 0.022 vs. OLETF_C; ^*D*^*P* = 0.026 vs. LETO and *P* = 0.004 vs. OLETF_C; ^*E*^*P* < 0.001 vs. LETO; ^*F*^*P* = 0.009 vs. LETO; ^*G*^*P* = 0.001 vs. LETO; ^*H*^*P* = 0.010 vs. LETO; ^*I*^*P* = 0.011 vs. LETO, *P* = 0.001 vs. OLETF_L and *P* = 0.001 vs. OLETF_V; ^*J*^*P* = 0.032 vs. LETO; ^*K*^*P* < 0.001 vs. LETO and *P* = 0.001 vs. OLETF_V; ^*L*^*P <* 0.001 vs. LETO and *P* = 0.030 vs. OLETF_V; ^*M*^*P* = 0.015 vs. LETO; ^*N*^*P* = 0.002 vs. LETO; ^*O*^*P* = 0.009 vs. OLETF_C; ^*P*^*P* = 0.006 vs. LETO and *P* = 0.021 vs. OLETF_V; ^*Q*^*P* = 0.006 vs. LETO and *P* = 0.021 vs. OLETF_V; ^*R*^*P* < 0.001 vs. all other groups; ^*S*^*P* = 0.002 vs. LETO; ^*T*^*P* = 0.010 vs. LETO; ^*U*^*P* = 0.002 vs. LETO.*

**FIGURE 1:**
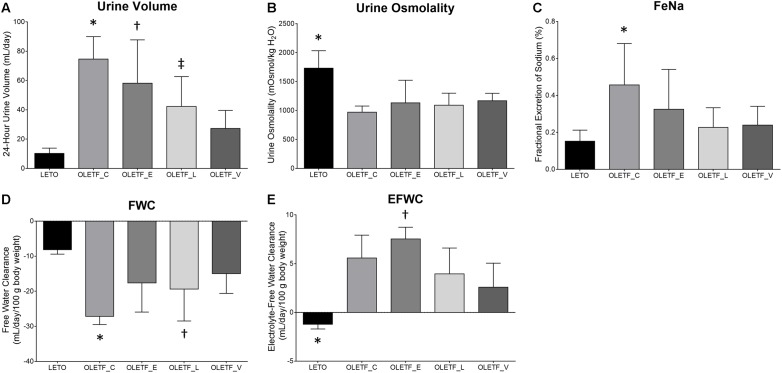
Empagliflozin-treated diabetic rats have high urine volume with slightly increased free water clearance. **(A)** Twenty four-hour urine output was significantly increased in untreated OLETF and empagliflozin- or lixisenatide-treated OLETF rats compared with LETO rats. ^∗^*P* < 0.001 vs. LETO; *P* = 0.027 vs. OLETF_L; *P* = 0.001 *vs.* OLETF_V. ^†^*P* < 0.001 vs. LETO. ^‡^*P* = 0.029 vs. LETO. **(B)** Urinary osmolality was significantly lower in all OLETF groups than in LETO group. ^∗^*P* < 0.0001 vs. OLETF_C; *P* = 0.009 vs. OLETF_E; *P* = 0.001 vs. OLETF_L; *P* = 0.010 vs. OLETF_V. **(C)** FeNa was increased only in the untreated OLETF group compared to the LETO group. ^∗^*P* = 0.007 vs. LETO. **(D)** FWC was significantly lower in untreated OLETF or lixisenatide-treated OLETF group compared to the LETO group. ^∗^*P* < 0.001 vs. LETO; *P* = 0.025 vs. OLETF_V. ^†^*P* = 0.020 vs. LETO. **(E)** EFWC was significantly higher in all OLETF groups compared to the LETO group, and administration of empagliflozin was associated with significantly higher EFWC compared with lixisenatide or voglibose. ^∗^*P <* 0.001 vs. OLETF_C; *P* < 0.001 vs. OLETF_E; *P* = 0.005 vs. OLETF_L; *P* = 0.026 vs. OLETF_V. ^†^*P* = 0.050 vs. OLETF_L and OLETF_V. *n* = 8 per each group.

In empagliflozin-treated OLETF rats, there was a marginal and non-significant decrease in urine volume compared with untreated OLETF rats (Figure 1A). The urine osmolality of all untreated and treated OLETF rats was significantly smaller than that of LETO rats, and no significant differences in urine osmolality were observed among the four diabetic rats (Figure 1B). Rats with untreated diabetes displayed increased 24-h urinary Na excretion compared with non-diabetic control (Table 1). There was a tendency of having less FeNa in all treated OLETF groups than in untreated OLETF group (Figure 1C), but this was not statistically significant. Without any treatment, diabetes mellitus in OLETF rats resulted in significant hyponatremia, whereas empagliflozin treatment of diabetic rats had no significant effect on serum Na level (Table 1). Untreated diabetes mellitus caused a marked reduction of FWC compared to rats that were not diabetic (Figure 1D). FWC of the empagliflozin-treated group showed no significant difference compared with the LETO and OLETF_C groups. Empagliflozin treatment resulted in an elevation of EFWC in OLETF rats compared with lixisenatide or voglibose treatment (Figure 1E).

Untreated OLETF and empagliflozin-treated OLETF rats displayed increased urinary excretion of K, compared with LETO and voglibose-treated OLETF rats (Table 1). However, FeK was significantly increased only in untreated OLETF rats, compared with LETO and voglibose-treated OLETF rats (Supplementary Figure S1A). TTKG was significantly lower in all OLETF groups than LETO group (Supplementary Figure S1B). In the voglibose-treated OLETF group a significant increase in serum Ca level, urinary Ca excretion and FeCa was observed (Table 1 and Supplementary Figure S1C). Serum P level did not differ among all groups, but there was an increase in urinary P excretion in empagliflozin-treated OLETF rats compared with the LETO group (Table 1), and FeP in the empagliflozin-treated OLETF group was significantly increased compared to the LETO and lixisenatide-treated OLETF groups (Supplementary Figure S1D). CCr was similar in all groups (Supplementary Figure S1E).

### Renal SGLT2 Expression in Diabetic Rat Kidneys Treated With Empagliflozin, Lixisenatide, or Voglibose

**FIGURE 2:**
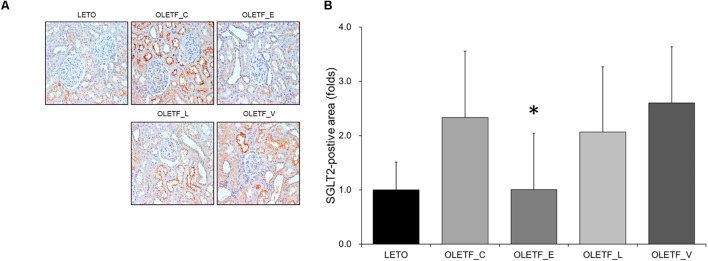
Empagliflozin decreases renal expression in SGLT2 in diabetic rat kidneys. **(A)** Representative renal sections immunostained with anti-SGLT2. **(B)** Quantitative analysis of results for SGLT2. ^∗^*P <* 0.001 vs. OLETF_C, OLETF_L, and OLETF_V. Magnification, ×200. *n* = 8 per each group.

The finding of immunostaining showed that empagliflozin treatment resulted in a decrease in SGLT2 expression in diabetic rat kidneys (Figure 2).

### Changes in Na Transport in Diabetic Rat Kidneys Treated With Empagliflozin, Lixisenatide, or Voglibose

**FIGURE 3:**
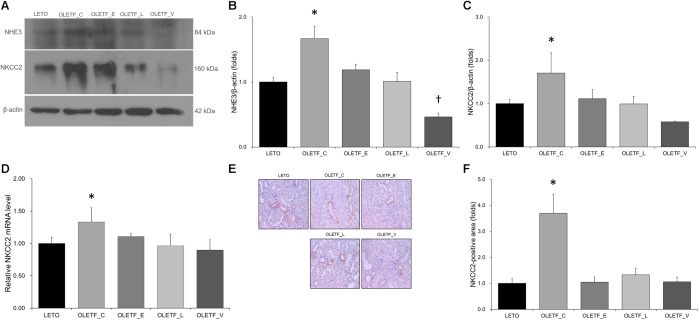
Empagliflozin tends to repress the increase in NHE3 and NKCC2 expression in diabetic rat kidneys. **(A)** Representative immunoblot reacting with anti-NHE3 and anti-NKCC2. **(B)** Densitometric analysis shows that renal NHE3 expression increased by diabetes mellitus is inhibited by treatment with lixisenatide or voglibose. ^∗^*P* = 0.008 vs. LETO; *P* = 0.016 vs. OLETF_L; *P* < 0.001 vs. OLETF_V; ^†^*P* < 0.000 vs. OLETF_E. **(C)** Protein expression of NKCC2 increased by diabetes mellitus is inhibited by treatment with empagliflozin, lixisenatide, or voglibose. ^∗^*P* = 0.013 vs. LETO; *P* = 0.033 vs. OLETF_E; *P* = 0.012 vs. OLETF_L; *P* < 0.001 vs. OLETF_V. **(D)** qRT-PCR shows that mRNA level of NKCC2 was elevated in untreated OLETF rats but it was unaffected with lixisenatide or voglibose treatment. ^∗^*P* = 0.024 vs. LETO; *P* = 0.019 vs. OLETF_L and OLETF_V. **(E)** Representative renal sections immunostained with anti-NKCC2. **(F)** Quantitative analysis of results for NKCC2 demonstrates the increased expression of NKCC2 in untreated diabetic kidneys. ^∗^*P* < 0.001 vs. other groups. Magnification, ×200. *n* = 8 per each group.

Compared with untreated or empgliflozin-treated OLETF, renal NHE3 expression was lower in voglibose-treated OLETF rats (Figure 3A,B). Protein expression of NKCC2 was significantly increased in the untreated OLETF group, and was decreased to a level similar to that of LETO group in all treated OLETF rats (Figure 3A,C). qRT-PCR and immunostaining of NKCC2 revealed the similar result as immunoblotting analysis (Figure 3D–F).

**FIGURE 4:**
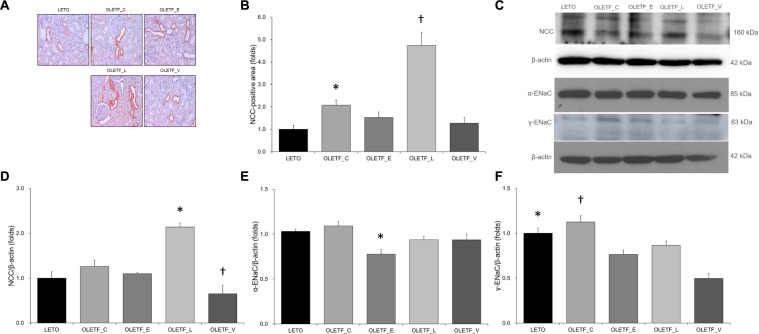
Empagliflozin does not significantly change NCC expression, but decreases ENaC expression in diabetic rat kidneys. **(A)** Representative renal sections immunostained with anti-NCC. **(B)** Quantitative analysis of results for NCC demonstrates the increased expression of NCC in lixisenatide-treated diabetic kidneys. ^∗^*P* = 0.004 vs. LETO. ^†^*P* < 0.001 vs. other groups. Magnification, ×200. **(C)** Representative immunoblot reacting with anti-NCC, anti-α-ENaC and anti- γ-ENaC. **(D)** Densitometric analysis shows that expression of NCC was significantly elevated in lixisenatide-treated OLETF rats. **^∗^***P* < 0.0001 vs. other groups. ^†^*P* = 0.019 vs. LETO; *P* = 0.002 vs. OLETF_C. **(E)** Densitometric analysis shows that abundance of α-ENaC was decreased in empagliflozin-treated diabetic rats compared with normal or diabetic control groups. ^∗^*P* = 0.035 vs. LETO; *P* = 0.009 vs. OLETF_C. **(F)** On densitometric analysis, expression of γ-ENaC, increased by untreated diabetes mellitus, was significantly decreased with all treatments. ^∗^*P* < 0.001 vs. OLETF_V. ^†^*P* = 0.006 vs. OLETF_E; *P* = 0.048 vs. OLETF_L; *P* < 0.001 vs. OLETF_V. *n* = 8 per each group.

The results of immunostaining and immunoblotting for NCC showed that lixisenatide treatment resulted in an increase in NCC (Figure 4A–D). Rats with empagliflozin-treated diabetes mellitus displayed decreased renal protein expressions of α-ENaC and γ-ENaC compared with untreated OLETF rats (Figure 4C,E,F).

### Effects of Empagliflozin, Lixisenatide, or Voglibose on Expression of Water Channels in Diabetic Rat Kidneys

**FIGURE 5:**
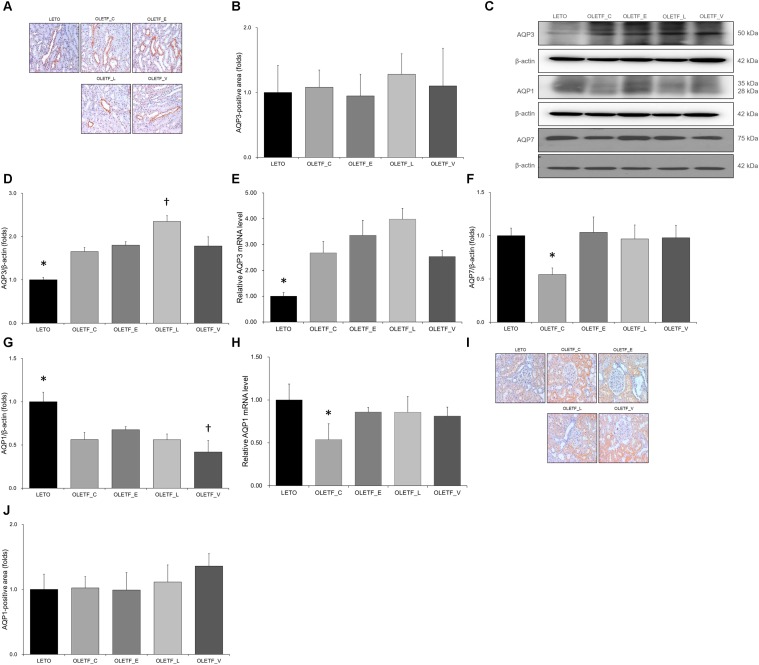
Empagliflozin causes no significant changes to AQP3 and AQP1 but tends to increase AQP7 level in diabetic rat kidneys. **(A)** Representative renal sections immunostained with anti-AQP3. **(B)** Quantitative analysis of results for AQP3 shows no significant change among groups. Magnification, ×200. **(C)** Representative immunoblot reacting with anti-NCC, anti-α-ENaC and anti- γ-ENaC. **(D)** Densitometric analysis shows that the expression of AQP3 protein significantly increased in all OLETF rats. Lixisenatide treatment further increased the expression of AQP3. ^∗^*P <* 0.001 vs. other groups. ^†^*P* < 0.001 vs. OLETF_C; *P* = 0.002 vs. OLETF_E; *P* = 0.003 vs. OLETF_V. **(E)** qRT-PCR shows that mRNA level of AQP3 significantly increased in all OLETF rats. ^∗^*P* = 0.001 vs. OLETF_C; *P* < 0.001 vs. OLETF_E and OLETF_L; *P* = 0.001 vs. OLETF_V. **(F)** Densitometric analysis shows that AQP7 in kidneys was significantly lower in untreated OLETF group than in LETO group and all treated OLETF groups. ^∗^*P* < 0.001 vs. LETO and OLETF_E; *P* = 0.001 vs. OLETF_L and OLETF_V. **(G)** Densitometric analysis shows the decreased AQP1 expressions in all OLETF rats. ^∗^*P* < 0.001 vs. other groups. ^†^*P* = 0.046 vs. OLETF_C; *P* < 0.001 vs. OLETF_E; *P* = 0.047 vs. OLETF_L **(H)** mRNA level of AQP1, decreased in untreated OLETF rats, was increased with antidiabetic treatment. ^∗^*P* < 0.001 vs. LETO; *P* = 0.003 vs. OLETF_E; *P* = 0.001 vs. OLETF_L; *P* = 0.002 vs. OLETF_V. **(I)** Representative renal sections immunostained with anti-AQP1. **(J)** Quantitative analysis of results for AQP1 shows no significant changes among groups. Magnification, ×200. *n* = 8 per each group.

In immunostaining for AQP3, no significant differences were observed among groups (Figure 5A,B). Both protein and mRNA levels of AQP3 were significantly increased in all OLETF groups (Figure 5C–E). Renal expression of AQP7 protein was significantly decreased in the untreated OLETF group and was restored to a level similar to that of LETO group by treatment with all antidiabetics (Figure 5C,F). The protein expressions of AQP1 were decreased in all OLETF groups (Figure 5C,G), and the AQP1 mRNA level in untreated OLETF rats was lower than those in other groups (Figure 5H). There were no significant differences were observed in immunostaining for AQP1 among groups (Figure 5I,J).

**FIGURE 6:**
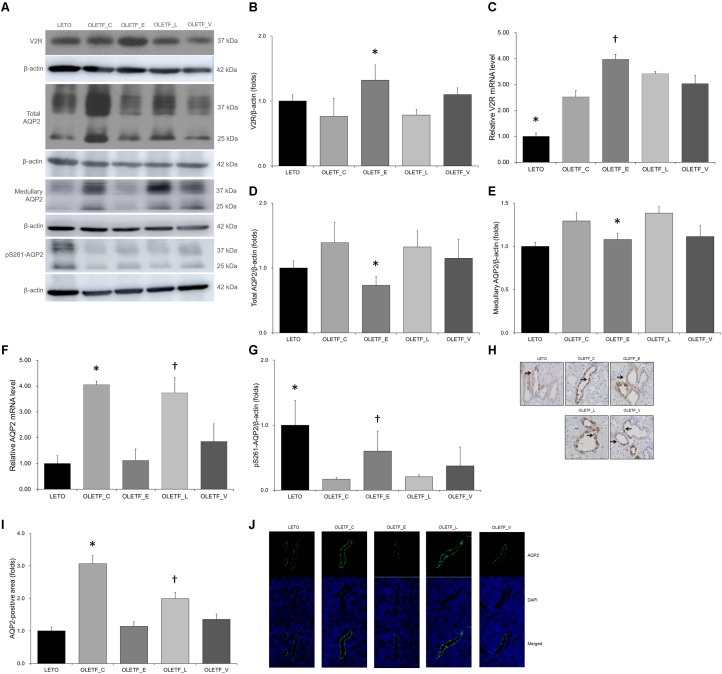
Empagliflozin increases V2R expression but decreases AQP2 expression in diabetic rat kidneys. **(A)** Representative immunoblot reacting with anti-V2R, anti-AQP2 and anti-p261-AQP2. **(B)** Densitometric analysis shows that renal expression of V2R protein in the empagliflozin-treated OLETF group was significantly increased compared with untreated OLETF or lixisenatide-treated OLETF rats. ^∗^*P <* 0.001 vs. other groups. **(C)** qRT-PCR shows that mRNA level of V2R was significantly increased in all OLETF rats than in LETO rats. Empagliflozin further increased V2R mRNA level compared with untreated OLETF control. ^∗^*P* = 0.001 vs. OLETF_C; *p* < 0.001 vs. OLETF_C, OLETF_L and OLETF_V. ^†^*P* = 0.041 vs. OLETF_C. **(D)** Protein expression of whole renal AQP2 was more decreased in empagliflozin-treated diabetic rats than in untreated or lixisenatide-treated diabetic rats. ^∗^*P* = 0.026 vs. OLETF_C; *P* = 0.045 vs. OLETF_L. **(E)** Medullary AQP2 expression was significantly decreased in empagliflozin-treated OLETF rats compared with control or lixisenatide-treated OLETF rats. ^∗^*P* = 0.035 vs. OLETF_C; *P* = 0.014 vs. OLETF_L. **(F)** mRNA level of renal AQP2 was significantly higher in untreated OLETF control and lixisenatide-treated OLETF rats. ^∗^*P* < 0.001 vs. LETO; *P* = 0.003 vs. OLETF_E. ^†^*P* < 0.001 vs. LETO and OLETF_E; *P* = 0.040 vs. OLETF_V. **(G)** The renal expression of pS261-AQP2 was significantly increased with empagliflozin treatment compared with no treatment or lixisenatide treatment in diabetic rats. ^∗^*P <* 0.001 vs. OLETF_C and OLETF_L. ^†^*P* = 0.004 vs. OLETF_C; *p* = 0.007 vs. OLETF_L. **(H)** Representative renal sections immunostained with anti-AQP2. **(I)** In quantitative analysis of results for AQP2, empagliflozin-tread OLETF rats have lower distribution of AQP2 protein in apical membranes of kidneys. ^∗^*P* < 0.001 vs. other groups. ^†^*P* < 0.001 vs. LETO and OLETF_E; *P* = 0.002 vs. OLETF_V. Magnification, ×200. **(J)** Representative immunofluorescence images show that the distribution of AQP2 in medullary area was relatively lower in empagliflozin- or voglibose-treated OLETF group than in other groups. Magnification, ×400. *n* = 8 per each group.

Renal expression of V2R protein was significantly higher in empagliflozin-treated OLETF rats than that in untreated (Figure 6A,B). The mRNA level of V2R was also significantly increased in the kidneys of empagliflozin-treated OLETF rats compared with untreated OLETF control rats (Figure 6C). Although renal V2R was upregulated, AQP2 protein expression in the whole kidney or medulla was significantly lower in empagliflozin-treated OLETF group compared with untreated or lixisenatide-treated OLETF group (Figure 6A,D,E). The mRNA level of AQP2 was consistently decreased by empagliflozin treatment (Figure 6F). Voglibose-treated OLETF rats also displayed lower AQP2 levels in their kidneys. The phosphorylation level of AQP2 at serine 261 was increased with empagliflozin treatment in diabetic rat kidneys (Figure 6A,G). Relatively lower AQP2 distribution was observed in kidneys of empagliflozin- and voglibose-treated OLETF rats (Figure 6H–J).

### Changes in Phosphorylation of p38-MAPK and Expressions of Phosphatase 2B and Cyclin-Dependent Kinases in Diabetic Rat Kidneys

**FIGURE 7:**
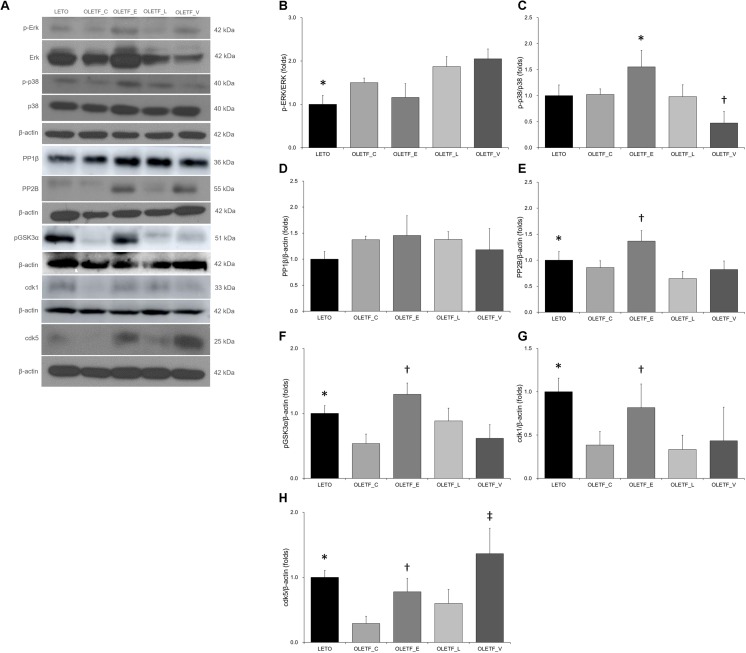
Empagliflozin leads to increase in the phosphorylation of p38-MAPK and expressions of phosphatase 2B and cyclin-dependent kinases in diabetic rat kidneys. **(A)** Representative immunoblot reacting with anti-p-Erk, anti-Erk, anti-p-p38, anti-p38, anti-PP1β, anti-PP2B, anti-pGSK3α, anti-cdk1 and anti-cdk5. **(B)** There was no significant change in renal protein ratio of p-ERK to ERK among diabetic rats. ^∗^*P* = 0.039 vs. OLETF_L; *P* = 0.005 vs. OLETF_V. **(C)** Empagliflozin treatment increased p-p38/p38-MAPK in diabetic kidneys. ^∗^*P* = 0.005 vs. LETO; *P* = 0.031 vs. OLETF_C; *P* = 0.004 vs. OLETF_L; *P* < 0.001 vs. OLETF_V. ^†^*P* = 0.009 vs. LETO; *P* = 0.022 vs. OLETF_E; *P* = 0.012 vs. OLETF_L. **(D)** There was no change in PP1β expression of non-diabetic or diabetic rat kidneys. **(E)** Empagliflozin significantly increased the PP2B abundance in diabetic rat kidneys. ^∗^*P* = 0.037 vs. OLETF_L. ^†^*P* = 0.028 vs. LETO; *P* = 0.002 vs. OLETF_C; *P* < 0.001 vs. OLETF_L; *P* = 0.001 vs. OLETF_V. **(F)** The renal expression of pGSK3α was significantly increased with empagliflozin treatment in diabetic rat kidneys. ^∗^*P <* 0.001 vs. OLETF_C; *P* = 0.010 vs. OLETF_V. ^†^*P <* 0.001 vs. OLETF_C and OLETF_V; *P* = 0.003 vs. OLETF_L. **(G)** The renal expression of cdk1 was significantly increased in empagliflozin-treated diabetic rats. ^∗^*P* = 0.001 vs. OLETF_C; *P <* 0.001 vs. OLETF_L; *P* = 0.002 vs. OLETF_V. ^†^*P* = 0.019 vs. OLETF_C and OLETF_V; *P* = 0.005 vs. OLETF_L; *P* = 0.046 vs. OLETF_V. **(H)** The renal expression of cdk5 was significantly increased in empagliflozin- or voglibose-treated diabetic rats. ^∗^*P* = 0.002 vs. OLET_C. ^†^*P* = 0.043 vs. OLETF_C. ^‡^*P <* 0.001 vs. OLET_C and OLETF_L; *P* = 0.006 vs. OLETF_V. *n* = 8 per each group.

It has been reported that phosphorylation of AQP2 at S261 by p38-mitogen-activated protein kinase (MAPK) contributes to controlling the protein level of AQP2 (Nedvetsky et al., 2010). In our experiment, empagliflozin increased protein level of p38 but not ERK (Figure 7A–C).

There was a previous report showing the possible involvement of PP1 or PP2 on AQP2 (Tamma et al., 2014). Immunoblotting study revealed that empagliflozin significantly increased the PP2B abundance but not PP1β (Figure 7A,D,E). The GSK3α level was also increased in empagliflozin-treated diabetic kidneys (Figure 7A,F). It has also been known that cdk1 and cdk5 phosphorylate AQP2 peptides at S261 (Tamma et al., 2014). We observed that cdk1 and 5 were increased with empagliflozin treatment (Figure 7A,G,H).

## Discussion

In this study, we observed the effects of a 12-week treatment of empagliflozin in diabetic OLETF rats with respect to diuresis, urine osmolality, excretion of electrolytes and minerals, and Na and water transport along the nephron. Empagliflozin-treated OLETF rats produced a slightly decreased but still high urine volume. Serum Na and K concentrations and FeNa did not significantly differ compared with untreated OLETF rats, indicating that water diuresis might also be responsible for polyuria in diabetic rats treated with empagliflozin. Furthermore, empagliflozin treatment in diabetic rats tended to further increase EFWC. This could be attributed mainly to osmotic diuresis from glycosuria by the inhibition of SGLT2. However, AQP2 abundance was downregulated at 12 weeks in the empagliflozin-treated rats despite the increased expression of V2R. Thus, long-term administration of empagliflozin could disrupt water reabsorption via AQP2 and increase non-glycosuric free water diuresis, contributing partially to polyuria.

The antidiuretic hormone (vasopressin)-V2R-AQP2 water channel axis is crucial. Dysregulation of vasopressin-induced AQP2 has been implicated in many clinical disorders of water homeostasis (Kim et al., 2015). In general, in conditions of water deprivation vasopressin is released in the blood circulation faster than it is synthesized (Hus-Citharel et al., 2014). Vasopressin acts on V2R in the collecting duct to increase water permeability in that duct, which is the major site for regulation of renal water handling (Hus-Citharel et al., 2014; Mamenko et al., 2016). The vasopressin signaling network between vasopressin stimulation and AQP2 trafficking to the apical plasma membrane has been identified (Kwon et al., 2013). Vasopressin promotes synthesis and trafficking of the AQP2 water channel to the apical membrane of the principal cells in the collecting duct (Mamenko et al., 2016). Because decreased abundance of AQP2 in the kidney is sufficient to produce the urinary concentrating defect (Khositseth et al., 2017), the inability to adequately respond to augmented vasopressin-V2R axis can result in production of large volumes of dilute urine, such as occurs in nephrogenic diabetes insipidus (Mamenko et al., 2016). Diabetes mellitus induces the upregulation of AQP2, even in the absence of vasopressin, to prevent excessive water loss (Kim et al., 2004; Cipriani et al., 2012; Thomson et al., 2012). In our study, AQP2 mRNA and protein were increased in untreated diabetic rats, while upregulation of V2R was evident in kidneys after 12 weeks of empagliflozin treatment, which suggests a disruption of signaling between V2R and AQP2 synthesis. These results indicate the possibility that empagliflozin treatment could be associated with AQP2 downregulation and subsequent development of mild and partial nephrogenic diabetic insipidus. This inappropriate AQP2 downregulation may be explained by the increased phosphorylation of S261 of AQP2 via p38-MAPK, cdk1/5, and GSK3α through PP2B as well as the decreased transcription of AQP2, shown by our results.

We also observed a significant change in expression of several renal Na transporters and channels in empagliflozin-treated diabetic rats compared with untreated diabetic controls. Increased urinary glucose excretion associated with SGLT2 inhibition might result in a development of electrolyte disorder (Chen et al., 2016). Consistent with a previous study (Chen et al., 2016), untreated diabetic rats had significant hyponatremia, while empagliflozin-treated diabetic rats did not develop dysnatremia. Increased excretion in the total amount of urinary Na and K was observed in untreated diabetic rats compared with non-diabetic rats, whereas empagliflozin-treated diabetic rats showed slightly decreased, but still high, excretion of urinary Na. Although there was a trend toward a decrease of FeNa and FeK with empagliflozin treatment, these values were not different from those of LETO control rats or untreated OLETF rats. The slight decrease FeNa with empagliflozin treatment is likely explained by the decrease in serum Na level as a result of osmotic fluid addition to the extracellular compartment during hyperglycemia (Rondon-Berrios et al., 2017). Interestingly, we found that most Na transporters and channels including NHE3, NKCC2, NCC, and ENaC were not upregulated and rather tended to decrease despite possible salt delivery in diabetic rats treated with empagliflozin. This would be the mechanism by which the natriuretic effect is partly maintained by empagliflozin. A previous study also observed a trend toward a decrease of NKCC2 with dapagliflozin treatment of diabetic rats (Chen et al., 2016). These observations run counter to the observation of increased NKCC2 protein production in diabetic rats (Kim et al., 2004). Increased expression of NKCC2 may play a role in enhancing the countercurrent multiplication system in the thick ascending limb (Kim et al., 2015). So, decreased expression of NKCC2 would interrupt water retention from the collecting duct. Given that the contribution of SGLT2 to apical Na uptake is quantitatively minor, with <5% of Na uptake along the proximal convoluted tubule being mediated by SGLT2 (Esteva-Font et al., 2012; Palmer and Schnermann, 2015), the effect of SGLT2 inhibition on Na transport along the whole nephron might not be substantial.

Another interesting finding was that NCC expression was upregulated only in lixisenatide-treated OLETF rats. The GLP-1 receptor is expressed in the brush border microvilli of proximal tubules (Rieg et al., 2012). Many gut-derived hormones including glucagon, secretin, and GLP-1 are natriuretic, and GLP-1 and its receptor agonist inhibit proximal tubular reabsorption and induce diuresis and natriuresis (Tanaka et al., 2017). In Wistar rats, intravenous infusion of GLP-1 for 60 min increases glomerular filtration rate, inhibits proximal tubular reabsorption, and increases urine flow and Na excretion (Crajoinas et al., 2011). Another study reported the preserved acute natriuretic effect of intravenous and/or intraperitoneal application of the GLP-1 receptor agonist, exendin-4, in a mouse model of type 2 diabetes mellitus (Rieg et al., 2012). Although the mechanisms by which GLP-1 induces diuresis and natriuresis are not completely understood, previous studies support the view that GLP has a role in downregulation of NHE3 activity in the renal proximal tubule (Crajoinas et al., 2011; O’Neill et al., 2011; Rieg et al., 2012). Presently, chronic administration of lixisenatide increased 24-h urine volume in diabetic rats compared with non-diabetic rats, and downregulated NHE3 expression. However, FeNa did not increase after the 12-week treatment with lixisenatide. This may be explained in part by the presently observed upregulation of NCC. Therefore, urine output that was increased by long-term administration of lixisenatide cannot be explained only by a natriuretic effect involving modulation of NHE3 activity.

In addition, there are notable findings that need to be further addressed. First, empagliflozin-treated rats showed an increase in urinary P excretion with compared with LETO rats and an increase in FeP compared with LETO or lixisenatide-treated rats. These findings are contrast to previous reports provided by manufacturer or advisory committee data concerning canagliflozin and dapagliflozin (Taylor et al., 2015). In our study, we observed slightly increased serum P in OLETF rats treated with empagliflozin. This supports the cautiously speculation that hyperphosphaturia could occur as a response reflecting an excess of serum P, probably released from bone, which is filtered by glomeruli and incompletely reabsorbed by tubuli. There is currently no evidence that SGLT inhibitors decrease tubular reabsorption of P (Taylor et al., 2015). Although a recent meta-analysis revealed no increased risk of bone fracture among patients with type 2 diabetes mellitus treated with SGLT2 inhibitors, a decline in bone mineral density and alteration of bone turnover markers have been reported (Ruanpeng et al., 2017). It is still necessary to evaluate the clinical effect on bone metabolism of SGLT2 inhibitors. Another unexpected finding was that long-term administration of voglibose resulted in hypercalcemia and hypercalciuria. These events may activate the Ca sensing receptor expressed in apical membranes in inner medullary collecting duct and enhance basal autophagy, resulting in autophagic degradation of AQP2 and subsequently contributing to development of nephrogenic diabetic insipidus (Khositseth et al., 2017). Consistent with this, our data showed that voglibose treatment lowered renal AQP2 levels. A previous report has indicated that hypercalciuria often occurs in diabetic individuals and is closely linked to glycemic control and hypovitaminosis D due to the decline in renal function (Montagnani et al., 2011).

Considering the multiple feedback systems involved over different timescales, interaction with the circulatory system, the complex organization of the tubule region of the nephron, and the heterogeneity of the renal epithelial cells (Desrochers et al., 2014; Hallow and Gebremichael, 2017), it would be difficult to separate the role or influence of other renal components in this study. In addition, it may not be easy to affirm that changes of sodium and water transporters/channels shown in this study are from direct or indirect effects of empagliflozin. Interestingly, the insulin-dependent action and extra-metabolic benefits of SGLT2 inhibitors, including reduction of albuminuria, fibrosis, oxidative stress and inflammation in diabetic or obese rat kidneys, have been recently suggested although the mechanism underlying other renal effects beyond their SGLT2 inhibition remains unexplained (Lee et al., 2018; Manne et al., 2019). Thus, the possibility that the pleiotropic effect of SGLT2 inhibitors affects renal tubules and interstitium cannot be excluded. The results of this study may offer clues to the mechanism underlying diuresis induced by long-term administration of empagliflozin in diabetic patients by measuring clinical factors, water channels, and Na transporters that can affect homeostasis of water and salt balance. Diuretic or aquaretic-based decongestion strategies have failed to improve long-term definite cardiovascular outcome (Felker et al., 2015). The possible mechanisms responsible for cardiovascular protection with SGLT2 inhibition may be attributed to its persistent diuretic and aquaretic effects as well as natriuretic ability.

## Conclusion

In conclusion, the collective data support the suggestion that diuresis induced by the long-term use of empagliflozin is likely to reflect glucose-driven osmotic diuresis and non-glycosuric free water diuresis, as well as natriuresis. In diabetic rat kidneys, the long-term use of empagliflozin may alleviate the upregulation of Na transporters, decrease total AQP2 expression and increase the phosphorylation of AQP2 at S261 through activation of p38-MAPK, PP2B and GSK3α, and cdk1 and cdk5 regardless of the upregulation of V2R. The long-term effect of empagliflozin in human for its natriuretic or aquaretic effects need to be further determined.

## Data Availability

All datasets generated for this study are included in the manuscript and/or the Supplementary Files.

## Author Contributions

SC, S-HK, and H-SK contributed to study design. SK, MS, MK, ESK, SJS, S-HK, and H-SK conducted the experiments and analyzed the data. SC, S-HK, and H-SK prepared the manuscript. All authors reviewed the manuscript.

## Conflict of Interest Statement

The authors declare that the research was conducted in the absence of any commercial or financial relationships that could be construed as a potential conflict of interest.
