# Supplementary material for: Empagliflozin Contributes to Polyuria via Regulation of Sodium Transporters and Water Channels in Diabetic Rat Kidneys
Source: Front Physiol. 2019 Mar 19;10:271. doi: 10.3389/fphys.2019.00271 (PMC6433843; doi:10.3389/fphys.2019.00271)
Supplement: Supplementary file 1 [file Data_Sheet_1.docx]

Supplementary Material

Empagliflozin Contributes to Polyuria via Regulation of Sodium Transporters and Water Channels in Diabetic Rat Kidneys

Sungjin Chung^1^, Soojeong Kim^2^, Mina Son^1^, Minyoung Kim^1^, Eun Sil Koh^1^, Seok Joon Shin^1^, Seung-Hyun Ko^3^,* and Ho-Shik Kim^2^,*

*** Correspondence:**

Seung-Hyun Ko

[kosh@catholic.ac.kr](mailto:kosh@catholic.ac.kr) or

Ho-Shik Kim

[hoshik@catholic.ac.kr](mailto:hoshik@catholic.ac.kr)

# Supplementary Figures


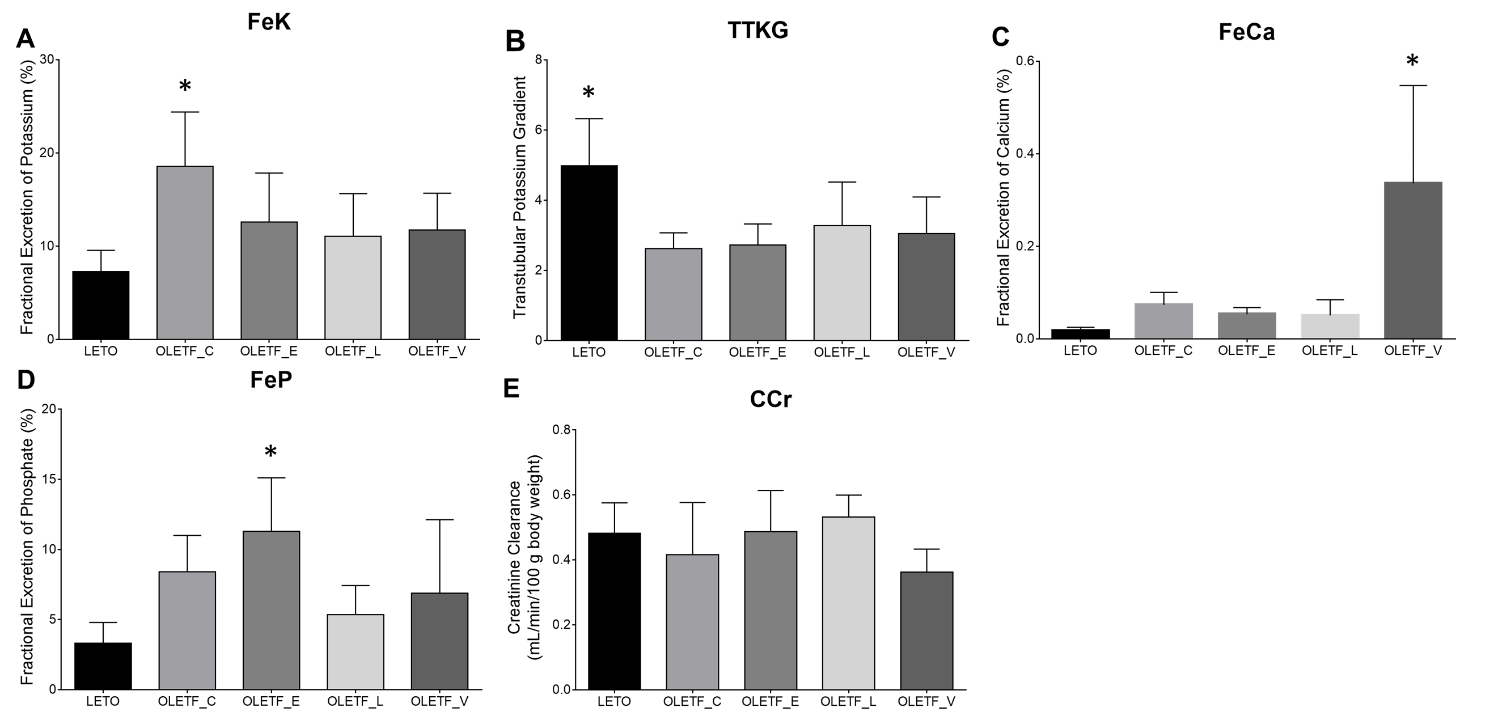


**Supplementary Figure 1.** Empagliflozin may affect fractional excretion of phosphate in diabetic rats. **(A)** Untreated OLETF rats had higher FeK than LETO rats or lixisenatide-treated OLETF rats. * *P=*0.001 *vs.* LETO; *P=*0.037 *vs.* OLETF_L. **(B)** TTKG was significantly lower in all OLETF groups compared with the LETO group. * *P=*0.001 *vs.* OLETF_C and OLETF_E; *P=*0.024 *vs.* OLETF_L; *P=*0.015 *vs.* OLETF_V. **(C)** FeCa was significantly increased in voglibose-treated OLETF rats compared with other groups. * *P* <0.001 *vs.* other groups. **(D)** FeP was significantly higher in empagliflozin-treated OLETF rats than LETO rats or lixisenatide-treated OLETF rats. * *P* <0.001 *vs.* LETO and *P*=0.009*vs.* OLETF_L. **(E)** There was no significant difference in creatinine clearance among the groups. *n*=8 per each group.
